# UBC4: A Repurposed Drug Regimen for Adjunctive Use During Bladder Cancer Treatment

**DOI:** 10.3390/biomedicines13030706

**Published:** 2025-03-13

**Authors:** Richard E. Kast

**Affiliations:** IIAIGC Study Center, 11 Arlington Ct, Burlington, VT 05408, USA; richarderickast@gmail.com

**Keywords:** celecoxib, melatonin, carbonic anhydrase, fluoxetine, ramelteon, repurposing

## Abstract

After it has metastasized, bladder cancer, the malignant transformation of the bladder urothelium, continues to be a common cause of death after maximal use of all currently available standard treatments. To address this problem in 2025, the drug repurposing movement within oncology aims to identify medicines in common general medical care use that have data indicating that they can interfere or inhibit a growth driving element that has been identified in bladder cancer. This paper now outlines extensive preclinical data showing that four drugs from general medical practice meet these criteria—the melatonergic drug ramelteon, the antidepressant fluoxetine, the antibiotic dapsone, and the analgesic drug celecoxib. This is the UBC4 regimen, meant as a possible adjunct added to standard treatments of metastatic bladder cancer. Three factors justify a clinical pilot trial of UBC4: (1) the UBC4 drugs are usually well tolerated and carry a low risk of harm, (2) the commonly fatal outcome of bladder cancer once it has widely metastasized, plus (3) the strong preclinical database showing UBC growth inhibition by each of the individual UBC4 drugs as outlined in this paper.

## 1. Introduction

This paper presents the details and rationale behind UBC4, a proposed new adjunctive drug regimen designed to be used alongside current standard treatments for urothelial bladder cancer (UBC). The current standard treatment of metastatic UBC includes platinum based chemotherapy, immune checkpoint focused inhibition, and anti nestin 1 monoclonal antibody [1A].

A section of the oncology community, physicians, and researchers, believe that while there is no silver bullet, a multidrug approach will be required for the long-term control or cure of the common metastatic cancers [1,2,3,4,5,6,7,8,9,10,11,12]. A multidrug approach is required, because like a well-designed complex machine, catastrophic malfunction usually does not arise from just one or two failed components. Several elements and redundant systems must simultaneously fail for the machine to fail. In aviation, this is called “the Swiss cheese model” in that the holes of each slice must align, and each slice must be moved for the hole to be complete through many slices.

Fitting the analogy to cancer, even in the case of a specific mutation creating a central driver, that mutation is not by itself sufficient for malignancy. EGFR positive non-small cell lung cancer, for example, is where initially, EGFR inhibitors allow tumor regression, but eventually, the cancer grows independently from EGFR. By the time the common deadly metastatic cancers are clinically recognized, multiple checks against metastasis and poorly unrestrained growth have been abrogated. It is the current dogma among those calling for multidrug approaches that many of these nonfunctioning checks must be pharmacologically blocked, taking the place of the cancer driving faulty or absent checks [1,2,3,4,5,6,7,8,9,10,11,12]. Cells, normal or malignant, have many work-arounds, should one or two be blocked.

UBC is a tumor resulting from the malignant transformation of urothelium. It is a common cause of death. Tobacco use, exposure to VOCs (volatile organic compounds), alcohol use, processed meat consumption, and exposure to industrial metals and dyes are common and well-known risk factors for UBC [13]. Common sites of UBC metastases are the bone, lung, and lymph nodes [14]. UBC4 is designed to be UBC subclass agnostic.

The UBC4 regimen uses the melatonergic drug ramelteon, the antidepressant fluoxetine, the antibiotic dapsone, and the analgesic drug celecoxib, as adjuncts, not replacements, to standard treatments of metastatic bladder cancer. Each of these drugs has a robust preclinical research database showing that they can inhibit UBC’s growth. Most of the preclinical work showing that is in vitro work with all the entailing caveats. Some of these drugs’ mechanisms of action in UBC growth inhibition are known and recounted here. Much of the preclinical work of UBC growth inhibition has been empirical.

Table 1 shows the suggested target doses for the UBC4 medicines. These doses are the average doses used in the respective drug’s day-to-day use in non-oncology general medical practice.

The UBC4 drugs were chosen by study of all repurposed drugs that have any published evidence for growth inhibition in UBC. Then, four drugs were selected for inclusion in UBC4 based (1) first on safety. Drugs without low side effect risks were not considered further. (2) The second criterion was the strength of preclinical evidence for UBC growth inhibition. Drugs with weak evidence were not considered further. (3) Having a strong and clear physiological rationale was the third selection criterion. (4) Then, among the few remaining drugs, clinical experience with a given drug favored its inclusion and little clinical experience with a drug favored exclusion. The four drugs remaining constitute UBC4; details follow.

## 2. The Drugs: Ramelteon, Fluoxetine, Celecoxib, Dapsone

### 2.1. Melatonin and Ramelteon

**Figure 1:**
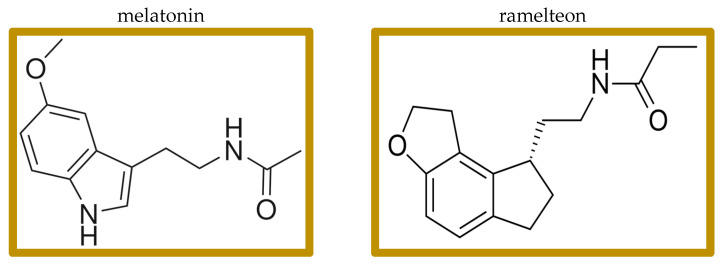
Structures of melatonin and ramelteon.

Melatonin is a simple, low-molecular-weight signaling molecule (Figure 1) with pleiotropic functions in all major body systems (pleiotropic—having “exceptional multiplicity of actions”) [15]. It is traditionally thought of as a hormone produced by the pineal gland, and indeed the pineal gland is responsible for most of the circulating blood melatonin; but melatonin is actually synthesized in and used for paracrine signaling by most body organs and tissues, where both primary receptors, M1 and M2, are also present [15,16,17,18,19,20]. Melatonin, traditionally thought of as one of the entraining elements directing diurnal rhythms, also has immune system effects [21,22,23]. Melatonin is available without a prescription in many jurisdictions worldwide. Many melatonin preparations have variable, poorly controlled contents [24]. It is, therefore, important to use only USP certified preparations.

The brain location of M1 differs from the M2 brain locations. Mice lacking MT1 have increased non-REM sleep but decreased REM sleep. Mice lacking MT2 have decreased in non-REM. In humans, structured dreams tend to be temporally associated with periods of REM, while illogical, bizarre dreams tend to occur in NREM. M1 and M2 heterodimers and M2 heterodimers with serotonin 5-HT2C receptors can form. Serotonergic agonism at 5-HT2C results in M2 signaling in absence of any melatonergic agonist [25,26].

Of uncertain physiological significance, melatonin also binds to several of the retinoic acid nuclear transcription factors.

Given this pleiotropy of melatonin effects and our lack of clearly definable melatonin actions, the dataset referenced below comes predominantly from empirical studies. A problem in interpreting experimental melatonin results derives from the lack of information on melatonin acting via its receptors, in which case, we can expect ramelteon to have similar effects, or melatonin acting via its physiological effects of the molecule itself, independent from its receptors, in which case, we might not expect ramelteon to have similar effects. Clinical results of melatonin have been inconsistent but do indicate a modest effect in promoting sleep.

A preclinical study showed that melatonin induced growth arrest in UBC cells [27,28]. Over a hundred studies have shown growth inhibition by melatonin in murine experimental tumors and in vitro cancer cell cultures [29,30,31,32,33]. No clinical study has yet replicated these results in humans.

NK cells are a subset of large granular lymphocytes that bear CD56, CD16, CD161, and lack CD3 expression [34]. Unlike most other T cells, their activation and target cell killing does not depend on antigen presentation. They synthesize and secrete IFN-gamma, perforin, granzyme B, and TNF. CD56 and CD16 surface marker expression: CD56 bright CD16 dim/low; these cells are less cytotoxic but secrete proinflammatory cytokines upon stimulation. CD56 dim CD16 high; this highly cytotoxic subset dominates the peripheral blood and plays a major role in NK-mediated tumor cell killing and metastasis prevention. Senescence and other signaling ligands that target cells for destruction by NK cells are usually absent in non-transformed cells but commonly become expressed on cells of a cancer. NK cells tend to be dysfunctional and are poorly activatable in UBC [35,36].

Adding exogenous melatonin increased the number, proliferation, degranulation, and IFN-gamma secretion of NK cells in aging mice [37]. The particularly high melatonin levels in bone marrow do not enter the general circulation in large amounts [17]. Exogenous melatonin increased the number and function of NK cells in old mice, a finding independently confirmed many times [37,38,39,40]. Melatonin induced a similar increased number and function of NK cells in ovariectomized rats [41]. NK cell number increased also in healthy young mice after prolonged oral melatonin supplementation [42]. Three mg oral melatonin lessened NK cell reduction in hemodialysis patients [43].

Oral melatonin slightly but statistically significantly increased baseline IL-2 in children with Down’s syndrome [44]. Increased IL-2 was seen in lupus mice given melatonin [45].

A 2012 meta-analysis of eight randomized controlled clinical trials of adjunctive melatonin treatment of cancer by Wang YM et al. included seven studies from the same single institution, with similar authors. None of the individual authors are answering emails of inquiry. The individual studies in that meta-analysis were of such poor quality that no conclusions of melatonin’s effectiveness can be made other than if it has any effect, it must be minor [46]. The most recent clinical study of melatonin in cancer was a study from 2014 in non-small cell lung cancer that showed no clinical effect on growth [47]. By itself, we should expect the same from adding single agent melatonin.

Ramelteon is an FDA approved generic non-scheduled melatonergic drug marketed to treat insomnia. Ramelteon has affinity for M1 and M2 receptors 3–16 times higher than that of melatonin and has a longer half-life than melatonin [48,49,50]. Clinical results of ramelteon have been inconsistent but do indicate a modest effect in promoting sleep.

Ramelteon increases 100-fold if co-administered with the antidepressant fluvoxamine [51]. Only three studies looked specifically at ramelteon as a melatonin agonist for cancer treatment adjunct. Nanomolar range ramelteon inhibited endometrial cancer cell growth, an effect blocked by a melatonergic receptor antagonist [52]. In vitro colon cancer cell growth was inhibited by ramelteon [53]. A review of the physiology of ramelteon’s potential for the inhibition of glioblastoma was collected and the evidence accrued up until 2015 was discussed [54].

A summary of the data on melatonin and ramelteon: we have evidence for the benefits as cancer treatment adjuncts. The evidence is weak but not zero. Since both melatonin and ramelteon are unlikely to have harms, bothersome side effects, or cancer growth enhancing effects, they are worth a try as an adjunct to other treatments.

### 2.2. Fluoxetine

Fluoxetine is the first selective serotonin reuptake inhibitor, marketed first in the late 1980s and is still in wide use to treat a depressed mood or anxiety [55,56]. In recent years, a developing research database has prompted interest in using adjunctive fluoxetine as part of a cancer treatment independent of its mood effects [57,58,59,60]. Table 2 lists some representative research evidence for fluoxetine’s inhibition of growth across a variety of different cancers.

Two studies suggest that UBC inhibition may be a class effect of SSRIs. Other SSRI class drugs, sertraline, and paroxetine also inhibited UBC growth in the 10 micromolar range [104]. A large population epidemiology study showed a decreased risk of developing UBC in those taking SSRIs fluoxetine, paroxetine, or citalopram [61].

Fluoxetine inhibits in vitro UBC growth without inhibiting non-transformed bladder urothelial cells [62]. Fluoxetine inhibited UBC growth with a cytotoxicity that was additive with cisplatin [63].

Several concerns limit the enthusiasm of adding fluoxetine to cancer treatment:

- In vitro studies showing growth inhibition tended to use low micromolar concentrations. That level is higher than ideal. Usually nanomolar growth inhibition marks strong candidate drugs.
- We do not have a unified mechanism of fluoxetine’s action in cancer growth inhibition. Different researchers have documented different cancer growth driving elements inhibited by fluoxetine. Examples: ERK1/2 pathway inhibition, inhibition of c-Myc, drug efflux pump inhibition, inactivating STAT3 driven epithelial to mesenchymal transition, mTOR activation, NK cell increase, and others [64,65,86,93,99,105,106,107].

Two considerations counterbalance these concerns: (a) the excellent tolerability of fluoxetine and low risk of harm, and (b) fluoxetine concentrates in tissue at several times the levels seen in blood [108,109].

### 2.3. Celecoxib

Celecoxib is a COX-2 selective inhibitor that also inhibits multiple carbonic anhydrase (CA) isoforms [110]. It has been in wide use since the 1990′s as a safe and effective analgesic drug [111].

Interest in celecoxib as an adjuvant during cancer treatment is based on both these attributes [112,113,114,115,116,117].

In bladder urothelial cancer, CA IX and CA XII are elevated; greater elevations shorten survival and are associated with a higher malignancy grade [118,119,120,121,122,123].

Celecoxib inhibits CA isoforms II, IV, IX, and XII [117,124,125,126]. CA-IX and XII are transmembrane and CA-II is soluble.

As an example of celecoxib’s CA inhibition profile compared to the standard CA inhibitor acetazolamide, Sethi et al. [126] found the following:

- acetazolamide...IC50 at CA-II = 12, CA-IX = 25, CA-XII = 6;
- celecoxib…........IC50 at CA-II = 21, CA-IX = 16, CA-XII = 18.

**Figure 2:**
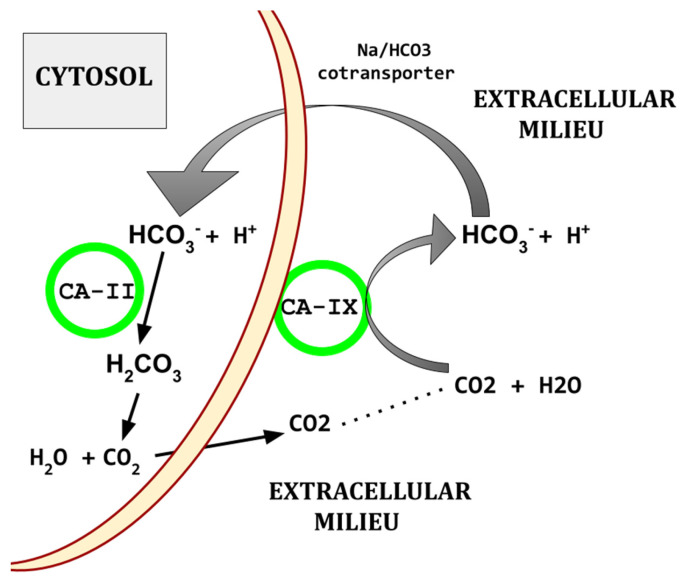
Diagram of basic CA based acid export. There are other paths by which protons are exported from cells.

Elevated CA isoforms are characteristic of UBC tissues [127]. CA-II and -IX regulate the pH of the UBC tumor microenvironment and are overexpressed in UBC. As illustrated in Figure 2, CA-II and CA-IX, working together, limit the lowering of intracellular pH that would otherwise result from the UBC hypermetabolic state.

Elevated CA isoforms -IX and -XII are a core feature also found across the common cancers [128,129,130,131,132,133]. Figure 2 shows the basic mechanism by which CAs participate in creating the characteristic acidic peritumoral milieu and maintenance of intracellular alkalinization.

Experimental CA-IX inhibitors inhibited UBC growth [134]. CA-IX is elevated in UBC tissue compared to normal urothelium, and the degree of elevation increases with tumor grade [120,135,136,137]. Urine CA-IX is elevated in UBC and correlates with UBC tissue CA-IX elevation [118,119,138]. Higher elevations of CA-IX expression in UBC tissue were associated with shorter survival and predict greater invasiveness and UBC’s metastatic potential [121,122,139]. Others found a UBC tissue elevation of CA-IX but no correlation with survival [140]. Serum carbonic anhydrase activity, subtype not specified, was elevated in UBC cases [141]. Urine exosomal mRNA for CA-IX is also elevated in UBC [142]. The reference pan-carbonic anhydrase inhibitor acetazolamide inhibited UBC growth [143].

As examples, CA-IX and/or CA-XII are elevated, worsen survival, and contribute to malignancy grade in many cancers. Examples: acute myelogenous leukemia [144,145], bladder urothelial cancer [118,119,120,121,122,123], breast [146], esophageal [147,148], gastric [148,149], glioblastoma [150], hepatocellular [151], Hodgkin’s lymphoma [152], laryngeal [153,154], nasopharyngeal [155,156], non-small cell lung [157], oral squamous cell [158], osteosarcoma [159], pancreatic ductal [160,161,162], and thyroid [163].

This dataset leads to the conclusion that CA-IX and or CA-XII tend to be one of the many links in mediating malignant cell behaviors across many of the common cancers. This finding is, however, not universal. Examples of exceptions: in renal cell carcinoma, higher CA-IX expression predicts a better prognosis [163,164,165], and in prostate cancer, CA-IX is absent [166,167].

Celecoxib, in adequate doses, may hobble UBC growth by limiting UBC’s ability to cope with its increased metabolism-related acid production. An indication that this is happening is the finding in glioblastoma, where resistance to temozolomide is overcome by CA-XII inhibition [168,169].

### 2.4. Celecoxib, IL-6

Of the many cytokines elevated in UBC, IL-6 plays an important if not central driving role. This section presents data, albeit indirect, suggesting that lowering IL-6 will improve UBC prognosis and that celecoxib will mediate that lowering.

Clinical use of celecoxib reduced the elevated circulating IL-6 found in patients with ankylosing spondylitis [170], pancreatitis [171], major depression [172], heavy tobacco smoking [173], frailty of age [174], knee osteoarthritis [175], and inflammatory arthritis [176].

IL-6 is the quintessential pleiotropic cytokine. Its signaling is ubiquitous and central to mammalian physiology. But what it does cannot be simply stated. IL-6 functions as a pro- or anti-inflammation element, depending on the circumstances [177,178]. In cancers generally, and in UBC specifically, IL-6 is predominantly growth promoting.

Excess IL-6 is one of the drivers of UBC growth, invasion, and migration. Oft repeated studies showed that serum or plasma IL-6 increases as UBC progressively invades the muscularis with higher IL-6 levels portending recurrence, metastasis, and shorter survival [179,180,181,182,183,184]. Urine IL-6 levels are elevated in UBC [179,185,186,187]. All clinical UBC biopsy cells expressed the IL-6 receptor [188].

IL-6 enhanced the in vitro growth of UBC cells [189]. In vitro inhibition of IL-6′s receptor inhibited UBC growth [182,190]. IL-6 enhances stem cell characteristics of UBC cells [191,192].

Serum IL-6 increases as UBC progressively invades the myometrium [179]. Urine IL-6 levels are elevated in UBC [179,185,187]. UBC cases with lymph node metastases have higher serum levels of IL-6 than do cases with low IL-6 [193].

The UBC cell subpopulation that exhibits stem cell characteristics of high ALDH expression, high clonogenicity, and low cell numbers needed to transplant the tumor have increased IL-6 receptor proteins compared to cells without these stem attributes [191].

### 2.5. Celecoxib and Tumor Resident Fibroblasts

Intratumoral, non-transformed fibroblasts of several different subcategories comprise a not insignificant percent of cells within the common cancers, UBC included. Their trophic function derives from providing structural support, and a wealth of growth and angiogenesis promoting factors [194,195,196,197]. Fibroblasts fulfill many crucial physiological functions crucial for the proper homeostatic functioning of all body organs: scaffolding, providing trophic growth signals, recruiting bone marrow cells for angiogenesis, shaping or inhibiting lymphocyte centered immune response, and others.

In UBC specifically, although essentially normal fibroblasts are enlisted to promote cancer growth, these UBC resident fibroblasts are responding normally to their normal activating, directing signaling systems. Their net effect promotes a pathology. Non-transformed, otherwise normal fibroblasts resident within UBC secrete higher levels IL-6 and IL-8 than normal, non-transformed bladder wall fibroblasts [198,199,200]. The trigger or signaling system driving these otherwise normal fibroblasts to secrete abnormal amounts of cancer trophic cytokines is unknown.

In vitro work has shown that celecoxib can diminish cancer tissue resident non-transformed fibroblasts’ trophic function to the malignant cell population [201,202,203]. IL-6 is one of several UBC resident fibroblasts’ growth enhancing products [204,205]. The COX-2 blocking function of celecoxib also contributes to the angiogenesis and fibroblast tumor trophic functions [206].

### 2.6. Dapsone, Neutrophils, and IL-8

Dapsone is one of the first of modern antibiotics, introduced to clinical practice in the 1940s, and is still in wide use. Dapsone is also active in treating or preventing Toxoplasmosis, Plamidia, Leishmania, and other protozoan infections. Dapsone’s use has expanded in the last two decades to include treatment of the neutrophilic dermatoses, bullous pemphigoid, or the EGFR inhibitor-induced rash as examples [207,208,209,210].

On that basis, dapsone’s use was further extended to other diseases and conditions characterized by the unwanted tissue destructive actions of neutrophil accumulations [210,211,212,213,214,215]. The further repurposing of dapsone on that same basis has potential to reduce the tumor trophic, and angiogenesis promoting functions of neutrophils during cancer treatment [3,4,5,11,216,217,218,219]. Dapsone impedes neutrophil chemotaxis along an IL-8 gradient. That is the basis for dapsone use in the neutrophilic dermatoses and during cancer treatment. By inhibiting neutrophils’ IL-8 chemotaxis, the tumor trophic, immunosuppressive neutrophil accumulations are reduced.

How exactly dapsone inhibits neutrophil chemotaxis along IL-8 gradients during the treatment of the neutrophilic dermatoses is not clear.

Neutrophils are absent in healthy bladder walls, but they heavily infiltrate UBC tissue where they appear to have a largely immunosuppressive role. UBC cells secrete CXCL1, CXCL5, and IL-8—all of which are chemotactic for neutrophils. Although preclinical research can demonstrate some tumor inhibiting properties, the preponderance of actual clinical evidence indicates a tumor trophic, growth enhancing, immunosuppressive role for neutrophils across the common human cancers [220,221,222,223].

Specifically, in UBC, higher tumor neutrophil infiltration portends shorter survival [224]. PGE2 synthesized by neutrophils’ COX-2 drives or contributes to the drive to UBC cells’ synthesis of immunosuppressive indoleamine 2,3-dioxygenase [224].

A metastatic UBC cell line synthesized more IL-8 than a corresponding non-transformed urothelium cell line. When that UBC cell line was treated with IL-8 full-sequence antisense cDNA, it made little IL-8 and lost the ability to metastasize [225]. An orthotopic UBC xenograft grew slower in mice given anti-IL-8 antibody [226]. Autocrine UBC cell IL-8 stimulates UBC cell migration/motility [227].

UBC cells synthesize and secrete IL-8, which is one of the primary drivers of neutrophil accumulations within UBC tissue [228]. UBC with stronger immunohistochemistry staining for IL-8 had more aggressive tumors and shorter survival [229,230]. Urine IL-8 levels are uniformly elevated in UBC and post-treatment elevated urine levels of IL-6 and IL-8 predicted UBC recurrence [118,138,187,230,231,232,233]. Margel et al. found 200 times greater urinary IL-8 in metastatic UBC compared to healthy controls [187]. Elevated serum levels of IL-8 in UBC also predicted shorter survival [230].

It is clear that neutrophils (a) play a predominantly immunosuppressive, tumor trophic role in UBC and (b) that excess IL-6 is one of the drivers of that [181,228,234,235]. Immune checkpoint blockade (pembrolizumab, nivolumab)-treated UBC patients with higher blood levels of IL-6 and IL-8 had poorer responses than did UBC patients with lower levels of these cytokines [236].

In UBC cases, as with other common cancers, a higher NLR correlates with higher IL-6 and IL-8 levels, higher IL-8 levels correlated with greater neutrophil infiltration into the tumor, and greater Treg numbers in UBC [237].

Of concern, and related to the preface of this paper, standard UBC cytotoxic chemotherapy increased the circulating and UBC tissue expression of IL-8 [238]. This makes adjunctive use of dapsone during standard cytotoxic chemotherapy of UBC particularly attractive.

### 2.7. TICO and the NLR

The ratio of neutrophils to lymphocytes in peripheral blood (NLR) is an interesting measure of prognosis across the common metastatic cancers. Confirmed in over one hundred clinical studies spanning the range of human cancers, a ratio >3:1 predicts poorer prognosis [239,240,241,242,243,244,245]. The remarkable finding of shorter survival in those with higher NLR across our common cancers reflects something profound: a unifying factor in cancers. We do not yet know what that unifying factor might be. Identified malignant tumor enhancing roles for neutrophils have been identified:

- Supplying VEGF and other factors contributing to angiogenesis;
- Inhibition of immune responses, becoming myeloid-derived suppressor cells;
- Contributions to peritumoral tissue destruction and preparation of tumor bed;
- Contributing tumor trophic factors.

There is no other marker or lab finding that is so uniformly, so often confirmed, and constantly seen across the entire range of human cancers than NLR elevation. Specifically in UBC, the NLR becomes elevated and a greater abnormal elevation reflects a more aggressive disease [246,247,248,249,250,251].

The TICO regimen is a four-drug regimen of repurposed drugs designed to lower the NLR [239]. TICO uses tadalafil, a phosphodiesterase 5 inhibitor used to treat pulmonary hypertension, isotretinoin, used to treat acne, colchicine, used to treat gout, and the fish oil omega-3. Unlike most repurposed drug regimens that are based on matching the pharmacology of a drug with the known growth pathways of a cancer, TICO is based on simple empirical observations of NLR lowering by these drugs. TICO has not been proven to lower the NLR in cancer. It is unlikely to harm or give any unpleasant side effects, so could be considered in UBC cases with elevated NLR > 3:1.

As mentioned in the section above on dapsone, it also has potential to lower the NLR [211,213].

## 3. Discussion

- In using repurposed drugs for the adjunctive treatment of cancer, patients and their doctors commonly underdose them. Repurposed drugs must be coordinated, and used at effective, usually robust doses [1,2,3,4,5]. The preclinical database supporting their use must be sound and many repurposed drugs must be used at the same time to progressively deplete cancer cells’ growth vigor as each individual drug is added [1,2,3,4,5].
- Celecoxib’s inhibition of neutrophils’ PGE2 plus dapsone’s reduction in tumor-resident neutrophils would operate together to decrease neutrophils’ tumor growth promotion.
- Drug levels used in many preclinical in vitro works documenting UBC growth inhibition were higher than levels we usually see during standard clinical use.
- Much—but not all—of the evidence showing UBC growth inhibition by the UBC4 medicines was performed in vitro. We have a long history of in vitro findings being not replicated when tried in the clinic. On the other hand, some of our current effective clinical treatments were originally demonstrated in vitro and they did translate well to the clinic.
- Melatonin products in the USA are poorly controlled. Some contain no melatonin; some contain other, non-listed hypnotic drugs [24]. If melatonin is used, it must be labeled USP.
- Dapsone use will generate some methemoglobinemia, usually <8%, and usually asymptomatic. The histamine receptor 2 inhibitor drug cimetidine will lower dapsone’s methemoglobinemia generation [252,253].
- Cimetidine dosed at 1400 mg q 12 h was well tolerated when treating papilloma verruca in immunosuppressed transplant recipients [254]. Nine studies prior to 2010 in various populations all reported significantly greater verruca resolution with cimetidine [255]. A common dose range for verruca treatment was 40 mg/kg/day.
- Science and medicine must cope with conflicting data. Except for the section on NLR, such is the case for UBC4. Datasets that guide medical practice usually have conflicting or unclear results in the early stages of their development.
- Rarely do clinicians have clear and unequivocal datasets supporting the adoption of a new treatment. Conflicting data in preclinical datasets are common. Fallacious data come about either by overt fabrication or by inadvertent errors of data generation or interpretation.
- Clinicians decide to adopt a treatment based on the preponderance of evidence, and the consideration of balancing risks of treatment versus risks of the target disease. This consideration reflects an old wisdom expressed in the myth of Scylla and Charybdis. As a metaphor, we face these two monsters today when confronting metastatic cancer. The Ionian poet Homer (~800 BCE) advises that we pass by Scylla, losing a few sailors eaten by Scylla rather than lose the entire ship swallowed up by the whirlpool of Charybdis.

Four common modes of failure when embarking on the use of repurposed medicines derive from the following: (a) the treating Oncologist declining to prescribe anything but the current standard chemotherapy for the given cancer; (b) the timidity and reluctance of the monitoring General Practitioner (Family Physician) to add multiple drugs; (c) the inadequate frequency of evaluations or monitoring for tolerability and adherence to a regimen that is unusually complicated; and (d) the use of repurposed drug doses that are too low to adequately inhibit the target system.

## 4. Conclusions

Much of the data presented here to support these four drugs having growth inhibiting effects in UBC have been performed in vitro. Most drugs with cancer inhibiting effects, in vitro or in rodent graft studies, eventually fail to benefit when tested clinically. Although this may turn out to be the case for UBC4, three considerations may warrant a pilot trial of UBC4: (a) good predicted tolerability and the unlikelihood of harm coming from the UBC4 drugs based on the wide past clinical experience with these drugs in their general medical indications, (b) the sound preclinical evidence of growth inhibition in UBC cells, and (c) the poor prognosis of metastatic UBC as things stand currently in 2025. 

## Figures and Tables

**Table 1:** Target daily doses for the UBC4 regimen. Except for celecoxib, these doses are at or near the usual doses used in general medical practice. For celecoxib, the listed dose is about twice the usual dose used to treat pain. See references on celecoxib in Section 2.3 below for the rationale behind that difference.

Drug	Target Dose
ramelteon	16 mg h
fluoxetine	40 mg × 1
celecoxib	600 mg × q 12 h
dapsone	100 mg × 1
cimetidine	800 mg q 8 h

**Table 2:** Representative studies reporting evidence for fluoxetine’s potential for reducing growth across a variety of different cancers. A common fluoxetine level in these in vitro culture studies was 10 to 40 microM. Fluoxetine concentrates in body tissues at 10 to 15 times greater levels than peak blood levels, typical levels ranging from 1 to 10 microg/mL in brain tissue.

Cancer Type	References	Evidence for Fluoxetine’s UBC Inhibition
bladder	[61,62,63]	in vitro, human epidemiological, additive with cisplatin
breast	[64,65,66,67]	in vitro, rodent graft
CLL	[68]	in vitro
colon	[69,70,71,72,73]	in vitro, rodent graft, additive with doxorubicin
gastric	[74,75,76]	in vitro, additive with paclitaxel
glioblastoma	[77,78,79,80,81]	in vitro, additive with temozolomide or irradiation
HCC	[82,83,84,85]	in vitro, rodent graft, additive with sorafenib
lymphoma	[86,87,88,89,90]	in vitro, immunoenhancement, rodent graft
melanoma	[91,92]	in vitro, rodent graft
myeloma	[90]	in vitro
lung, adeno	[83,93,94,95]	in vitro, rodent graft
medulloblastoma	[96]	in vitro
osteosarcoma	[97]	in vitro
ovarian	[98]	in vitro
pancreatic	[99,100]	in vitro, rodent graft, immunoenhancement
prostate	[101,102]	in vitro
rhabdomyosarcoma	[96]	in vitro
squamous cell	[103]	in vitro
